# Vision‐related quality of life after unilateral occipital stroke

**DOI:** 10.1002/brb3.3582

**Published:** 2024-07-02

**Authors:** Neil Dogra, Bryan V. Redmond, Selena Lilley, Brent A. Johnson, Byron L. Lam, Madhura Tamhankar, Steven E. Feldon, Berkeley Fahrenthold, Jingyi Yang, Krystel R. Huxlin, Matthew R. Cavanaugh

**Affiliations:** ^1^ Department of Ophthalmology, Flaum Eye Institute and Center for Visual Science University of Rochester Rochester New York USA; ^2^ Department of Biostatistics and Computational Biology University of Rochester Rochester New York USA; ^3^ Bascom Palmer Eye Institute University of Miami Miami Florida USA; ^4^ Scheie Eye Institute University of Pennsylvania Philadelphia Pennsylvania USA

**Keywords:** cortical blindness, hemianopia, quadrantanopia, quality of life, stroke

## Abstract

**Background/objectives:**

Stroke damage to the primary visual cortex induces large, homonymous visual field defects that impair daily living. Here, we asked if vision‐related quality of life (VR‐QoL) is impacted by time since stroke.

**Subjects/methods:**

We conducted a retrospective meta‐analysis of 95 occipital stroke patients (female/male = 26/69, 27–78 years old, 0.5–373.5 months poststroke) in whom VR‐QoL was estimated using the National Eye Institute Visual Functioning Questionnaire (NEI‐VFQ) and its 10‐item neuro‐ophthalmic supplement (Neuro10). Visual deficit severity was represented by the perimetric mean deviation (PMD) calculated from 24‐2 Humphrey visual fields. Data were compared with published cohorts of visually intact controls. The relationship between VR‐QoL and time poststroke was assessed across participants, adjusting for deficit severity and age with a multiple linear regression analysis.

**Results:**

Occipital stroke patients had significantly lower NEI‐VFQ and Neuro10 composite scores than controls. All subscale scores describing specific aspects of visual ability and functioning were impaired except for ocular pain and general health, which did not differ significantly from controls. Surprisingly, visual deficit severity was not correlated with either composite score, both of which increased with time poststroke, even when adjusting for PMD and age.

**Conclusions:**

VR‐QoL appears to improve with time postoccipital stroke, irrespective of visual deficit size or patient age at insult. This may reflect the natural development of compensatory strategies and lifestyle adjustments. Thus, future studies examining the impact of rehabilitation on daily living in this patient population should consider the possibility that their VR‐QoL may change gradually over time, even without therapeutic intervention.

## Graphical abstract

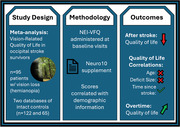

## 1 INTRODUCTION

Occipital stroke is the leading source of damage to the human primary visual cortex (V1; Zhang et al., 2006), causing a homonymous loss of conscious vision over portions of the visual field through both eyes (Gilhotra et al., 2002; Pollock et al., 2011, 2012). This condition is known by numerous names but in this manuscript, it will be referred to as cortically induced blindness (CB). It affects a significant portion of stroke survivors (Rowe et al., 2019), with an estimated 1% of the population over age 49 years likely to develop CB in their lifetime (Gilhotra et al., 2002), and ∼100,000 new cases each year in the United States and Europe (Gray et al., 1989; Pollock et al., 2011, 2012; Rowe, 2013; Sahraie, 2007).

Activities of daily living including reading, driving, navigation, and autonomy are severely impaired in CB (Dombovy et al., 1986; Jones & Shinton, 2006; Jongbloed, 1986). Until recently, this vision loss was considered irreversible, with most patients discharged without rehabilitation opportunities (Horton, 2005a, 2005b; Plant, 2005; Reinhard et al., 2005) and as a result, there is little follow‐up or monitoring of progression in CB. This is despite growing recommendations for rehabilitative options (Pollock et al., 2019; Willis & Cavanaugh, 2023) and accumulating experimental evidence that visual training can recover a range of perceptual abilities within CB fields (for review, see Liu et al., 2019; Melnick et al., 2016; Saionz et al., 2020, 2022). As restoration therapies targeting CB continue to develop, a better understanding of the spontaneous evolution of vision‐related quality of life (VR‐QoL) and the major factors driving these changes is essential to correctly interpret the impact of both the condition, and its rehabilitation, on daily living.

The National Eye Institute Visual Functioning Questionnaire (NEI‐VFQ) is a clinically validated, self‐administered survey that has been extensively used to evaluate VR‐QoL in CB patients (Chen et al., 2009; Gall et al., 2009, 2010; George et al., 2011; Papageorgiou et al., 2007; Rowe et al., 2019). It asks respondents to assess difficulties they face with vision‐specific functioning in contexts that include social gatherings, workplace performance, and pursuit of personal hobbies (Mangione et al., 1998, 2001). Twelve subscale scores are generated from these responses, as well as a composite score describing overall VR‐QoL. The NEI‐VFQ can also be administered with a 10‐item neuro‐ophthalmic supplement (Neuro10), which generates an independent, composite score describing neuro‐ophthalmic functions (Raphael et al., 2006).

One prior study found lower NEI‐VFQ composite scores in 33 patients with stroke‐induced V1 damage compared to a control group of healthy subjects (Papageorgiou et al., 2007). The authors reported that time since stroke and visual deficit severity did not affect NEI‐VFQ scores, but only patients in the chronic phase (≥6 months) poststroke were included (Papageorgiou et al., 2007). Similar findings were reported in another sample of 177 first‐ever, chronic stroke patients (Gall et al., 2010). Finally, a study that included both acute and subacute patients (*n* = 66) reported a large impact of deficit severity on VR‐QoL, but did not assess the effect of time poststroke (Tharaldsen et al., 2020). Here, we examined VR‐QoL in 95 CB patients with V1 damage from a single etiology (occipital stroke), and in the absence of any therapeutic intervention for their vision loss. Our goal was to assess if VR‐QoL differed for patients <1 month to several years poststroke. We also gauged the relative influence of deficit size/severity and participant age on this natural history. Finally, by examining individual subscale scores, we asked which aspects of visual or social functioning drove observed differences.

## 2 METHODS

### 2.1 Participants

**FIGURE 1:**
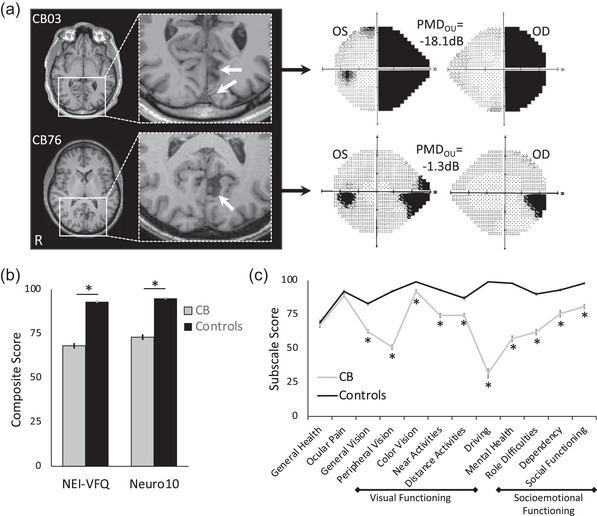
(a) Sample magnetic resonance images (T1) of two cortically induced blindness (CB) patients, whose left (oculus sinister [OS]) and right (oculus dextrus [OD]) eye Humphrey visual fields (HVFs) are shown adjacently. For the brain images, radiological convention is used, with the right brain hemisphere (R) on image left. White arrows on enlargements of the regions inside the boxes point to lesion site(s) in the occipital lobe of each patient. On HVFs, black shading denotes a sensitivity of 0 dB, whereas light stippling indicates higher visual sensitivities. Average, binocular (oculus uterque [OU]) perimetric mean deviations (PMDs) are indicated for each patient. Note that the larger brain lesion in CB03 gives rise to a larger area of HVF defect and more negative PMD, than the smaller brain lesion in CB76. (b) Mean National Eye Institute Visual Functioning Questionnaire (NEI‐VFQ) and 10‐item neuro‐ophthalmic supplement (Neuro10) composite scores comparing the present cohort of CB patients with previously published controls (Mangione et al., 2001; Raphael et al., 2006). Mean patient age in our CB cohort did not differ significantly. Controls attained significantly higher composite scores than CB patients on both measures. (c) Plot of individual NEI‐VFQ subscale scores CB patients and the same controls whose composite scores are shown in b. Unsurprisingly, controls scored higher for every subscale except for general health (*p* = 0.480) and ocular pain (*p* = 0.112). Scores evaluating visual functioning and socioemotional functioning are outlined, with “driving”—the most severely affected subscale—separating these two major categories. Error bars in b and c = standard errors of the mean. *p < 0.001.

We conducted a retrospective, meta‐analysis of VR‐QoL data from 95 patients with homonymous vision loss from stroke‐induced damage to the occipital cortex, confirmed by magnetic resonance imaging or computed tomography (Figure 1a). Patients were enrolled in one of two vision restoration clinical trials (ClinicalTrials.gov identifiers: NCT04798924, NCT03350919), or in experimental studies conducted by the Huxlin laboratory (NCT05098236). Exclusion criteria in all studies were: best‐corrected visual acuity worse than 20/40, concurrent use of medications that might affect test performance, presence of ocular or neurologic condition(s) other than occipital stroke, which might cause visual impairment or impede study performance. By including data from three different trials, we were able to include a larger number of patients and times poststroke than were present in individual studies.

VR‐QoL surveys were collected prior to and in the absence of training or rehabilitative interventions. CB patients (female/male = 26/69) were aged 27–78 (mean ± standard deviation = 58 ± 12) years old, and ranged from 0.5 to 373.5 (26.0 ± 55.4) months poststroke (see Supporting Information Table 1 for breakdown per NCT). Patient data were compared to two published reference groups of visually intact controls: one for the NEI‐VFQ and another for its Neuro10. The NEI‐VFQ reference group consists of 122 visually intact participants, 59 ± 14 years old (Mangione et al., 2001); the Neuro10 reference group includes 65 visually intact controls, 38 ± 12 years old (Raphael et al., 2006). Mean patient age in our CB cohort did not differ significantly from the NEI‐VFQ reference group (*t*_216 _= 0.56, *p* = 0.578), but the Neuro10 reference group was significantly younger (*t*_159 _= 10.38, *p* < 0.0001).

Clinical procedures for the present study were approved by the Western Institutional Review Board for patients enrolled in clinical trial NCT03350919 (WIRB#1181904) and by the Research Subject Review Board at the University of Rochester for NCT04798924 and NCT05098236. All procedures complied with the tenets of the Declaration of Helsinki and were conducted after receiving written, informed consent from each participant.

### 2.2 Quality of life measures

The questionnaires administered included the 25‐item version of the NEI‐VFQ along with its 14‐item appendix, resulting in a total of 39 questions. As per the scoring manual, responses from the NEI‐VFQ were used to compute 12 subscale scores: general health, general vision, ocular pain, near activities, distance activities, social functioning, mental health, role difficulties, dependency, driving, color vision, and peripheral vision. Subscale scores were then averaged together (excepting general health) to generate a composite score of overall VR‐QoL (Mangione, 2000). We also administered the Neuro10, which generated an independent Neuro10 composite score. All scores scale from 0 to 100, with higher scores indicating better functioning. Patients received a paper copy of the survey and were instructed to independently complete the questionnaire at our clinical testing sites. Study staff were present to clarify wording but provided no additional input or assistance.

### 2.3 Assessment of visual field defect size and severity

Relative size and severity of CB visual impairments were estimated using a Humphrey Visual Field Analyzer II‐i at two study sites (87 patients), and a Humphrey Visual Field Analyzer 3 at a third site (eight patients), with all sites using a 24‐2 testing protocol. Patients were presented with white, Goldman size III stimuli on a white background with a luminance of 11.3 cd/m^2^. Visual sensitivity thresholds for detecting these light targets were calculated using the Swedish Interactive Threshold Algorithm (SITA‐Standard). Visual acuity was best corrected to ≥20/40 using trial lenses, and fixation was controlled with gaze/blind spot automatic settings. Only reliable tests were included in our analysis, defined by fixation loss, false‐negative, and false‐positive rates <20%. Perimetric mean deviations (PMDs), which contrast a participant's visual field against age‐matched, visually intact controls, were calculated monocularly by a proprietary algorithm (Carl Zeiss Meditech), then averaged between eyes to generate a single composite value for visual deficit size/severity in each patient. More negative PMD values indicated greater visual deficit size/severity over the central 54° of the visual field.

### 2.4 Statistical analyses

Mean NEI‐VFQ subscale scores, composite scores, and Neuro10 scores were compared between CB patients and visually intact controls using unpaired *t*‐tests. Simple linear regressions were used to assess the linear relationships of time poststroke, age, and PMD with NEI‐VFQ and Neuro10 composite scores. Multiple linear regression analyses were used to determine the associations between outcomes—NEI‐VFQ subscale scores, NEI‐VFQ composite scores, and Neuro10 composite scores—and independent variables (i.e., time poststroke, PMD, and age) in CB patients in the presence of other risk factors. Analyses were conducted using statistical software (R Studio Version 2023). Statistical significance was set at *p* < 0.05.

## 3 RESULTS

### 3.1 Characteristics of visual deficits

The present cohort of CB patients presented a wide range of visual deficit sizes and severity, with PMDs ranging from −19.3 to −1.5 dB, with a mean of −9.9 ± 4.1 dB. Examples of a small and large deficit are shown in Figure 1a. Critically for subsequent analyses, PMD was not significantly correlated with patient age (*r* ^2 ^= 0.047, *p* = 0.113) or time since stroke (*r* ^2 ^= 0.062, *p* = 0.065), nor was there a significant correlation between time since stroke and patient age (*r* ^2 ^= 0.066, *p* = 0.162).

### 3.2 Impact of occipital stroke on VR‐QoL

In CB patients, the mean NEI‐VFQ composite score was 68.2 ± 15.3 and the mean Neuro10 score was 73.0 ± 15.8, both significantly lower than control participants, who scored 93.1 ± 6.8 on the NEI‐VFQ and 95.0 ± 5.0 on the Neuro10 (Figure 1b and Table 1). Individual NEI‐VFQ subscales were impaired on 10/12 categories (all but general health and ocular pain; Table 1 and Figure 1c) in CB patients, but interestingly, none were significantly correlated with PMD (Supporting Information Figure 1). Differences between CB patients and controls were greatest for driving, peripheral vision, mental health, and role difficulties (Table 1). With respect to driving, two CB patients reported that they stopped driving for nonvision‐related reasons. Per the NEI‐VFQ scoring manual, a driving score could not be generated for these patients; therefore, they could not be included in further analysis. Of the remaining CB patients, approximately 53% (49/93) reported giving up driving due to their vision, generating a driving score of zero, while the rest continued to drive.

**TABLE 1:** Descriptive statistics contrasting National Eye Institute Visual Functioning Questionnaire (NEI‐VFQ) and 10‐item neuro‐ophthalmic supplement (Neuro10) scores between cortically induced blindness (CB) patients and visually intact controls.

Scale	CB	Control	*t*	df	*p*
NEI‐VFQ composite score	68.2 ± 15.3	93.1 ± 5.0	−16	216	<0.0001*

General health	66.9 ± 18.5	69.0 ± 24.0	−0.7	216	0.48
General vision	62.6 ± 15.8	83.0 ± 15.0	−9.7	216	<0.0001*

Ocular pain	88.9 ± 15.7	92.0 ± 13.0	−1.6	216	0.112
Near activities	74.3 ± 19.1	93.0 ± 11.0	−9.6	216	<0.0001*

Distance activities	74.4 ± 16.9	87.0 ± 18.0	−11.6	216	<0.0001*
Social functioning	80.8 ± 18.8	98.0 ± 10.0	−8.7	216	<0.0001*

Mental health	57.2 ± 24.4	98.0 ± 8.0	−13	216	<0.0001*
Role difficulties	61.9 ± 23.7	90.0 ± 15.0	−10.7	216	<0.0001*

Dependency	75.6 ± 26.3	93.0 ± 13.0	−6.4	216	<0.0001*
Driving	31.7 ± 37.1	99.0 ± 6.0	−19.7	214	<0.0001*

Color vision	92.1 ± 15.2	99.0 ± 3.0	−4.9	216	<0.0001*
Peripheral vision	50.9 ± 20.5	92.0 ± 12.0	−18.5	216	<0.0001*
Neuro10 composite score	73.0 ± 15.8	95.0 ± 5.0	−10.9	159	<0.0001*

Control data were previously reported controls (Mangione et al., 2001; Raphael et al., 2006).

* denotes significance.

### 3.3 Relationship between NEI‐VFQ subscale scores

**FIGURE 2:**
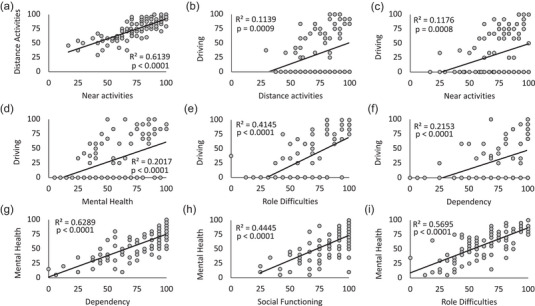
Significant intercorrelations between key visual functioning and socioemotional subscales of the National Eye Institute Visual Functioning Questionnaire (NEI‐VFQ). Plots of one subscale score against another with each data point denoting a single cortically induced blindness (CB) patient. (a) Simple regression analysis showed distance activities to be correlated significantly and positively with near activities. (b) Similarly, driving correlated significantly and positively with distance activities. (c) Driving correlated significantly and positively with near activities. (d) Driving correlated significantly and positively with mental health. (e) Driving correlated significantly and positively with role difficulties. (f) Driving correlated significantly and positively with dependency. (g) Mental health correlated significantly and positively with dependency. (h) Mental health correlated significantly and positively with social functioning. (i) Mental health correlated significantly and positively with role difficulties.

We used simple linear regressions to correlate subscale scores with one another. One of the strongest relationships occurred between scores for distance activities and near activities (Figure 2a). Driving was also significantly correlated with these two subscales (Figure 2b,c), but its strongest correlations were with mental health, role difficulties, and dependency (Figure 2d‐f). Lastly, scores describing socioemotional functioning (mental health, role difficulties, dependency, and social functioning) were most strongly intercorrelated with one another. Mental health was most significantly associated with role difficulties, dependency, and social functioning (Figure 2g‐i).

### 3.4 Evolution of QoL scores with time poststroke

**FIGURE 3:**
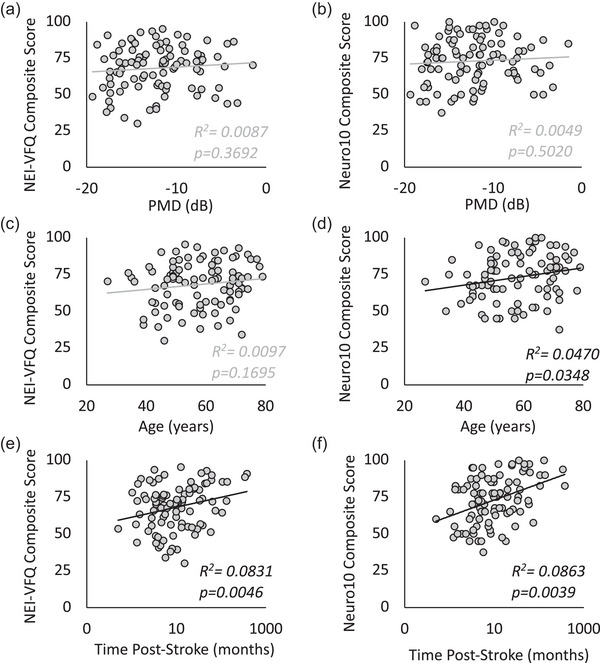
Simple linear regressions correlating age, perimetric mean deviation (PMD), and time poststroke individually with National Eye Institute Visual Functioning Questionnaire (NEI‐VFQ) and 10‐item neuro‐ophthalmic supplement (Neuro10) composite scores. Plots of composite scores against PMD, age, and time poststroke, with each data point denoting individual patients. PMD was not significantly correlated with NEI‐VFQ composite score (a) or Neuro10 composite score (b). Age was not significantly correlated with NEI‐VFQ composite score (c), but Neuro10 composite score increased significantly with age (d). Both NEI‐VFQ composite score (e) and Neuro10 score (f) increased significantly with time poststroke—relationships that were maintained after conducting multivariate regression analyses that considered PMD and age (see Table 2 for details).

In simple linear regressions, neither PMD nor age were significantly correlated with NEI‐VFQ composite scores (Figure 3a,b). PMD was also not correlated with Neuro10 composite scores (Figure 3c), but patient age was (Figure 3d). However, simple linear regressions showed both composite scores to increase with time poststroke (Figure 3e,f)—correlations that remained significant after adjusting for age and PMD with multiple linear regression analysis (Table 2). A 1‐month increase in time poststroke increased the average NEI‐VFQ and Neuro10 composite scores by ∼0.1 units each in this model (Table 2).

**TABLE 2:** Multivariate regression analyses of quality of life (QoL) scores and time poststroke, adjusted for perimetric mean deviation (PMD) and age.

Scale		Slope	Standard error	*t*	*p*
NEI‐VFQ composite score		0.09	0.03	3.01	.003*
	**General health**	**0.08**	**0.03**	**2.41**	.018*
	General vision	0.1	0.03	3.69	.0004*
	**Ocular pain**	**‐0.02**	**0.03**	**‐0.74**	**.459**
	Near activities	0.06	0.04	1.59	.116
	**Distance activities**	**0.08**	**0.03**	**2.5**	.014*
	Social functioning	0.04	0.03	1.28	.203
	**Mental health**	**0.14**	**0.04**	**3.08**	.003*
	Role difficulties	0.11	0.04	2.5	.014*
	**Dependency**	**0.11**	**0.05**	**2.29**	.025*
	Driving	0.19	0.07	2.71	.008*
	**Adjusted driving**	**0.11**	**0.05**	**2.21**	.033*
	Color vision	0.04	0.03	1.4	.165
	**Peripheral vision**	**0.07**	**0.04**	**1.88**	**.064**
Neuro10 composite score		0.08	0.03	2.94	.004*

* indicates significance.

Two participants were enrolled >300 months poststroke: their enrollment dates poststroke were each more than 300 months longer than the mean enrollment time poststroke (26.0 months) and both more than 325 months longer than the median enrollment time poststroke (9.1 months). To assess whether these two data points influenced the slope estimate in our linear regression models, that is, whether they acted as statistical leverage points, we repeated the multivariate regression excluding these two patients; both the NEI‐VFQ and Neuro10 composite scores still increased significantly with time poststroke (*p* = 0.034 and *p* = 0.0008, respectively).

Finally, adjusting for PMD and age, 7/12 NEI‐VFQ subscale scores improved with increasing time poststroke: general health, general vision, distance activities, mental health, role difficulties, dependency, and driving (Table 2), even when the analysis excluded participants who stopped driving poststroke (“adjusted driving” score, Table 2). The remaining five subscale scores did not change significantly with time since stroke: ocular pain, near activities, social functioning, color vision, and peripheral vision.

## 4 DISCUSSION

The present cross‐sectional analysis of postoccipital stroke patients adds to the growing discussion regarding the impact of visual field deficits on VR‐QoL. Ours is one of the first studies to include both subacute (<6 months poststroke) and chronic (>6 months poststroke) patients in the same analysis, while focusing solely on unilateral stroke sustained in adulthood. This allowed us to assess a wider range of times poststroke than previous work. Moreover, not excluding patients by deficit size or lesion age (Gall et al., 2010) allowed novel correlations to emerge between these parameters and VR‐QoL. Specifically, we uncovered that VR‐QoL appears to increase with time poststroke, irrespective of deficit size or patient age. Subscale analyses provided key insights into the likely drivers of this unexpected relationship.

### 4.1 Occipital stroke reduces vision‐related QoL

We compared VR‐QoL scores among our sample of CB patients to two reference groups of visually intact controls—one for the NEI‐VFQ and another for the Neuro10 supplement. The NEI‐VFQ control group is referenced extensively in studies quantifying the impact of ophthalmic diseases on VR‐QoL (Cahill et al., 2005; Clemons et al., 2008; Hariprasad et al., 2008; Ma et al., 2002; Schiffman et al., 2001). This group was well matched in age to our sample of CB patients, who nonetheless had significantly lower NEI‐VFQ composite scores. CB patients also scored lower than controls on the Neuro10 supplement, although the reference group for the latter was younger than our CB patients. Overall, reductions in both NEI‐VFQ and Neuro10 composite scores confirmed that VR‐QoL was impaired in our cohort of CB stroke‐only patients.

Notably, 24‐2 binocular Humphrey PMD, an objective proxy for central visual deficit size and severity, failed to correlate with NEI‐VFQ or Neuro10 composite scores when adjusting for age and time poststroke. Subscale analyses revealed a similar lack of correlation between PMD and all NEI‐VFQ subscales (Supporting Information Figure 1), suggesting that CB patients’ perceived difficulty in visual and socioemotional functioning is not related to the objective severity or size of their central visual deficit. This finding contrasts somewhat with prior literature reporting improved VR‐QoL scores with increased central visual field sparing (Gall et al., 2010; Papageorgiou et al., 2007), and greater improvement in VR‐QoL with greater spontaneous improvement in deficit size up to 6 months poststroke (Tharaldsen et al., 2020). Our results may instead reflect a greater contribution of other social functioning factors on VR‐QoL scores in the present cohort, or be due to our multivariate analyses, which accounted for both age and time since stroke.

One important consideration in the context of the present study is that while 24‐2 Humphrey visual field (HVF) perimetry is the most commonly used clinical tool in the United States for quantifying visual deficits in CB, it does not capture the entirety of the visual field and may thus miss some of the deficit. In future studies, quantification and categorization (hemianopia, scotoma, degree of macular sparing, etc.) of the CB deficit with whole‐field methods (e.g., Goldman, or automated kinetic perimetry using the Octopus) may address this problem and it is conceivable that they may yield stronger correlations with VR‐QoL than Humphrey perimetry. Finally, VR‐QoL in this patient population could be impacted by patient visual acuity. Presently, all patients were best corrected to 20/40 or better, and foveal acuity is not thought to be impacted in homonymous hemianopia, which typically presents with normal foveal sensitivity (as was the case in all of our patients’ HVFs). Finally, previous reports found no difference in VR‐QoL in occipital stroke patients when comparing patients with good versus poor central acuity (Gall et al., 2010).

### 4.2 Subscale‐specific impairments

When investigating the impact of occipital stroke on subscales of the NEI‐VFQ, CB patients scored significantly lower than age‐matched controls for each subscale except for general health and ocular pain, consistent with prior work (Mangione et al., 2001). The lack of impairment in these two subscales was unsurprising, as occipital stroke is not associated with physical discomfort to the eye, and CB patients enrolled met stringent medical and functional criteria to participate in the studies analyzed (see Section 2). In fact, our CB self‐reported general health scores were as good as those of healthy controls and suggest that differences in other subscales were due to the stroke's impact on vision, instead of other impairments seen in the general stroke population.

The observed impairments among CB patients for subscales describing visual ability (general vision, peripheral vision, near activities, and distance activities) were expected, since homonymous hemianopia is characterized by deficits that often span large segments of the visual periphery. Impairments in socioemotional subscales (mental health, role difficulties, dependency, and social functioning) may instead be related to poststroke depression, which has been documented in up to half of sufferers within the first 5 years poststroke (Ayerbe et al., 2014; Hackett & Pickles, 2014; Kim & Choi‐Kwon, 2000). Impairments in mental health and related socioemotional subscales may also be associated with the reduced ability to drive. Driving was the subscale where CB patients scored most poorly compared to controls, with 53% reporting that they gave up driving entirely due to their eyesight. Driving cessation has also been associated with increased depressive symptoms in a general population of older adults (Ragland et al., 2005). This was confirmed here, with driving scores most strongly correlated with mental health, role difficulties, and dependency scores, and driving, or lack thereof, may also be a determining factor in role fulfillment.

On‐road testing found CB patients to have noticeable, but not intractable, driving deficits. Chronic CB patients have at‐fault accident rates 2.6 times higher than the visually intact population, motivating driving restrictions for hemianopic patients (McGwin et al., 2016). However, on‐road testing found that up to 82% of drivers with homonymous hemianopia committed no obvious errors while driving (Elgin et al., 2010), and when evaluators were masked to the participants’ condition, 73% of hemianopic and 88% of quadrantanopic patients were deemed fit to drive (Wood et al., 2009). Given the large impact of driving on QoL, evaluation for driving safety and rehabilitation may offer a simple and cost‐effective method of improving CB patients’ QoL. In addition, a subset of patients may adapt well enough to their deficit to return to driving, regardless of intervention (Howard et al., 2022). Finally, we note that pass/fail results for on‐road testing and simulator environments show good fidelity in CB patients (Ungewiss et al., 2018), suggesting that simulated tests could provide a safe, naturalistic space for examination of CB driving ability (Bowers, 2016; Bowers et al., 2009).

### 4.3 VR‐QoL increases naturally with time since stroke

A key finding here was that VR‐QoL increases with time poststroke, even when adjusting for age and PMD. Our multivariate analysis included a much larger range of poststroke times (<1 month to over 31 years) than any prior work (Gall et al., 2009, 2010; Papageorgiou et al., 2007). Improvement in health‐related QoL with time was previously reported in the general stroke population, but occipital stroke patients showed slower recovery than their visually intact counterparts (Gall et al., 2010). A distinct possibility is that this slower rate of improvement is a feature of global QoL for CB patients, resulting from the lack of available rehabilitation compared to that for speech or motor impairments. Nonetheless, the gradual improvement in VR‐QoL over time offers hope for CB patients dealing with life changes in the early stages poststroke.

To understand what aspects of visual functioning drove the observed larger VR‐QoL at later times poststroke, we examined individual NEI‐VFQ subscale scores, adjusting for age and PMD. Seven improved significantly over time: general health, general vision, distance activities, mental health, role difficulties, dependency, and driving. It is notable that these scores were significantly correlated with each other in our subscale analyses. As such, any of these categories may drive the observed *overall* improvement, enhancing other categories in the process. Alternatively, they may improve independently, or with some mixed amount of driving effects from specific subscales. The remaining five subscales (ocular pain, near activities, social functioning, color vision, and peripheral vision) did not improve over time poststroke. Ocular pain was not impacted by this stroke, leaving near activities, social functioning, color vision, and peripheral vision as potential targets for rehabilitation and counseling.

In our earlier comparison with visually intact controls, we noted that mental health and role difficulties were among the most impaired subscales in CB patients, likely reflecting a loss of self‐sufficiency and professional ability. Increased scores for mental health, role difficulties, and dependency over time may reflect the gradual development of lifestyle adjustments that help patients contend with their visual impairment and recover some self‐sufficiency and professional functioning. Alternatively, patients may simply become accustomed to their deficit and “learn to live with it,” despite the burdens it imposes.

Finally, higher VR‐QoL scores at later times poststroke do not negate the fact that as a cohort, CB patients remained impaired relative to controls in all subscales excepting ocular pain and general health (Figure 1). Thus, accelerating recovery or enhancing its magnitude may be critical for ultimately improving patient outcomes.

### 4.4 Limitations

A key limitation of the present study was an inability to assess VR‐QoL in the same patient, over time. Instead, we relied upon the different times poststroke across individuals to make inferences about the evolution of VR‐QoL. It is hoped that future studies will address this problem and rule out the possibility of a sampling bias within the CB population who volunteers for research studies such as ours, long after their stroke.

Additionally, while the NEI‐VFQ was scored in this study according to suggested guidelines, this approach is not without flaws. Specifically, local dependence between items and subscales may confound interpretation of NEI‐VFQ outcomes (Pesudovs et al., 2010). New scoring methods have attempted to alleviate these issues (Goldstein et al., 2022) and may provide a more reliable and accurate assessment in CB. However, because we relied on published data for our visually intact control cohort, we were unable to employ these new methods.

We should also note that differences in age may have confounded comparison of Neuro10 scores between CB patients and controls, since the reference group for those scores was significantly younger than our CB patients (Raphael et al., 2006). However, given the extreme degree of VR‐QoL impairment in CB evidenced by the NEI‐VFQ, and its lack of correlation with age, it is likely our observations would hold for the Neuro10, even with appropriately matched controls. Related to this, concerns have been raised over the utility of the Neuro10 in cases of stroke‐induced vision loss, as several of the supplement's questions reference impairments that are unlikely to occur as a result of occipital stroke (Rowe et al., 2019). Thus, when the Neuro10 score is used as part of the calculation for the overall NEI‐VFQ composite score it may artificially inflate the outcome. This was not the case here, as we did not use the Neuro10 score as part of the NEI‐VFQ composite score calculation, and we found a significant impairment in Neuro10 scores for CB participants versus visually intact controls.

Finally, although the NEI‐VFQ has been used extensively to quantify VR‐QoL in CB patients, we note that it was originally designed for patients with ophthalmic disease (Gall et al., 2009). As such, it may not robustly capture all aspects of VR‐QoL that pertain to CB (Hepworth & Rowe, 2016). The recent development and validation of a CB‐specific questionnaire will likely significantly improve our understanding of this condition and its impact on daily life (Hepworth et al., 2019, 2023).

## 5 CONCLUSIONS AND IMPORTANCE OF KNOWLEDGE GAINED

VR‐QoL is significantly reduced by occipital strokes sustained in adulthood. Neither deficit size/severity nor age were significant predictors of this reduction. However, our cross‐sectional design and large range of times poststroke revealed that both composite scores and some key, individual subscores to be greater at later versus earlier times poststroke. This suggests VR‐QoL tends to increase with time, though repeating VR‐QoL assessment at multiple time points will be necessary to confirm this possibility, as individual differences significantly impact VR‐QoL. These results provide new, potentially key information for patients, clinicians, and researchers as to the main drivers of VR‐QoL after occipital stroke. It is now possible to describe what changes patients might expect as they progress in their stroke recovery. Just as importantly, it alerts the field to the fact that VR‐QoL may improve spontaneously after occipital stroke, a trend against which vision restoration therapies in this patient population should be measured in order to assess their true impact.

## AUTHOR CONTRIBUTIONS

**Neil Dogra**: Writing—original draft; methodology; data curation; formal analysis; visualization; conceptualization; investigation; writing—review and editing; validation. **Bryan V. Redmond**: Writing—original draft; writing—review and editing; investigation; formal analysis; data curation; methodology; validation; visualization. **Selena Lilley**: Writing—review and editing; data curation; formal analysis; investigation; methodology. **Brent A. Johnson**: Writing—review and editing; formal analysis; validation; visualization; investigation; methodology. **Byron L. Lam**: Writing—review and editing; supervision; project administration; methodology; conceptualization; investigation; resources. **Madhura Tamhankar**: Writing—review and editing; supervision; project administration; conceptualization; methodology; investigation; resources. **Steven E. Feldon**: Writing—review and editing; funding acquisition; resources; project administration; supervision; conceptualization; investigation; validation; methodology. **Berkeley Fahrenthold**: Writing—review and editing; methodology; data curation; investigation; validation; visualization; formal analysis. **Jingyi Yang**: Writing—review and editing; investigation; data curation; methodology. **Krystel R. Huxlin**: Conceptualization; writing—original draft; writing—review and editing; project administration; resources; supervision; funding acquisition; methodology; validation; visualization; investigation; formal analysis; data curation. **Matthew R. Cavanaugh**: Conceptualization; writing—original draft; writing—review and editing; supervision; project administration; formal analysis; software; data curation; investigation; visualization; validation; methodology.

## CONFLICTS OF INTEREST STATEMENT

The authors declare no conflicts of interest.

### PEER REVIEW

The peer review history for this article is available at https://publons.com/publon/10.1002/brb3.3582.

## Supporting information

Supporting Information Figure 1. Simple linear regressions correlating perimetric mean deviation (PMD) with key subscales describing visual functioning. (a) PMD was not significantly correlated with scores for distance activities. (b) PMD was not significantly correlated with scores for near activities. (c) PMD was not significantly correlated with scores for general vision. (d) PMD was not significantly correlated with scores for peripheral vision. (e) PMD was not significantly correlated with scores for driving.

Supporting Information Table 1. Demographic characteristics across the three NCT analyzed in the present study. Other than female (F) and male (M) numbers, all data are reported as mean ± SD.

## Data Availability

The data that support the findings of this study are available from the corresponding author upon reasonable request.
