# Correction to “Vision‐related quality of life after unilateral occipital stroke”

**DOI:** 10.1002/brb3.71390

**Published:** 2026-04-13

Dogra, N., Redmond, B. V., Lilley, S., Johnson, B. A., Lam, B. L., Tamhankar, M., Feldon, S. E., Fahrenthold, B., Yang, J., Huxlin, K. R., & Cavanaugh, M. R. (2024). Vision‐related quality of life after unilateral occipital stroke. Brain and Behavior, 14, e3582. https://doi.org/10.1002/brb3.3582

In re‐analyzing data in this article for a follow‐up study, we discovered that we incorrectly reported the order of visually‐intact control means and standard deviations for the NEI‐VFQ subscale scores extracted from a prior publication (Mangione et al., 2001) into our Figure 1C and Table 1. In addition, there was an error in the listed degrees of freedom (df) for the control subject comparisons in Table 1: they were listed as (n1+n2)‐1 instead of (n1+n2)‐2.

Fortunately, these errors do not change the presence/absence of statistical significance for any of the comparisons made between control data and our stroke patient data, nor the conclusions drawn from our study. As reported originally, all subscale and score differences are highly significant, except for ocular pain and general health.

Below is the revised Figure 1, with corrected control data for Panel C:

**FIGURE 1:**
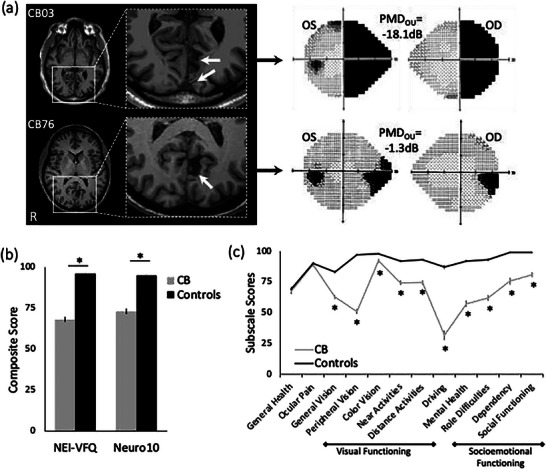
(A) Sample magnetic resonance images (T1) of two CB patients, whose left (OS) and right (OD) eye Humphrey visual fields (HVFs) are shown adjacently. For the brain images, radiological convention is used, with the right brain hemisphere (R) shown on the left side of the image. White arrows on enlargements of the regions inside the boxes point to lesion site(s) in the occipital lobe of each patient. On HVFs, black shading denotes a sensitivity of 0 dB, whereas light stippling indicates higher visual sensitivities. Average, binocular (OU) PMDs are indicated for each patient. Note that the larger brain lesion in CB03 gives rise to a larger area of HVF defect and more negative PMD than the smaller brain lesion in CB76. (B) Mean NEI‐VFQ and Neuro10 composite scores comparing the present cohort of CB patients with previously published controls (Mangione et al., 2001; Raphael et al., 2006). Mean patient age in our CB cohort did not differ significantly. Controls attained significantly higher composite scores than CB patients on both measures. (C) Plot of individual NEI‐VFQ subscale scores of CB patients and the same controls whose composite scores are shown in B. Unsurprisingly, controls scored higher for every subscale except for general health (*p* = 0.480) and ocular pain (*p* = 0.62). Scores evaluating visual functioning and socioemotional functioning are outlined, with “driving”—the most severely affected subscale—separating these two major categories. Error bars in B and C = standard errors of the mean. *p < 0.001.

Below is the revised Table 1 with corrected control means and SD for the NEI‐VFQ subscale scores, and corrected df for all comparisons:

**TABLE 1:** Descriptive statistics contrasting National Eye Institute Visual Functioning Questionnaire (NEI‐VFQ) and 10‐item neuro‐ophthalmic supplement (Neuro10) scores between cortically induced blindness (CB) patients and visually intact controls.

Scale	CB	Control	*t*	df	*p*
NEI‐VFQ composite score	68.2 ± 15.3	93.1 ± 5.1	−16.8	215	<0.0001^*^
General health	66.9 ± 18.5	69.0 ± 24.0	−0.7	215	0.48
General vision	62.6 ± 15.8	83.0 ± 15.0	−9.7	215	<0.0001*
Ocular pain	88.9 ± 15.7	90.0 ± 15.0	−0.5	215	0.62
Near activities	74.3 ± 19.1	92.0 ± 13.0	−8.1	215	<0.0001*
Distance activities	74.4 ± 16.9	93.0 ± 11.0	−9.8	215	<0.0001*
Social functioning	80.8 ± 18.8	99.0 ± 3.0	−10.5	215	<0.0001*
Mental health	57.2 ± 24.4	92.0 ± 12.0	−13.8	215	<0.0001*
Role difficulties	61.9 ± 23.7	93.0 ± 13.0	−12.3	215	<0.0001*
Dependency	75.6 ± 26.3	99.0 ± 6.0	−9.5	215	<0.0001*
Driving	31.7 ± 37.1	87.0 ± 18.0	−14.4	213	<0.0001*
Color vision	92.1 ± 15.2	98.0 ± 8.0	−3.7	215	0.0003
Peripheral vision	50.9 ± 20.5	97.0 ± 10.0	−21.7	215	<0.0001*
Neuro10 composite score	73.0 ± 15.8	95.0 ± 5.0	−10.9	158	<0.0001*

*Note*: Control data were previously reported (Mangione et al., 2001; Raphael et al., 2006). An asterisk (*) denotes statistical significance.

We apologize for these errors.

