# Supplementary material for: Vision‐related quality of life after unilateral occipital stroke
Source: Brain Behav. 2024 Jul 2;14(7):e3582. doi: 10.1002/brb3.3582 (PMC11219293; doi:10.1002/brb3.3582)
Supplement: Supplementary file 1 — Supporting Information Figure 1. Simple linear regressions correlating perimetric mean deviation (PMD) with key subscales describing visual functioning. (a) PMD was not significantly correlated with scores for distance activities. (b) PMD was not significantly correlated with scores for near activities. (c) PMD was not significantly correlated with scores for general vision. (d) PMD was not significantly correlated with scores for peripheral vision. (e) PMD was not significantly correlated with scores for driving. [file BRB3-14-e3582-s001.pdf]

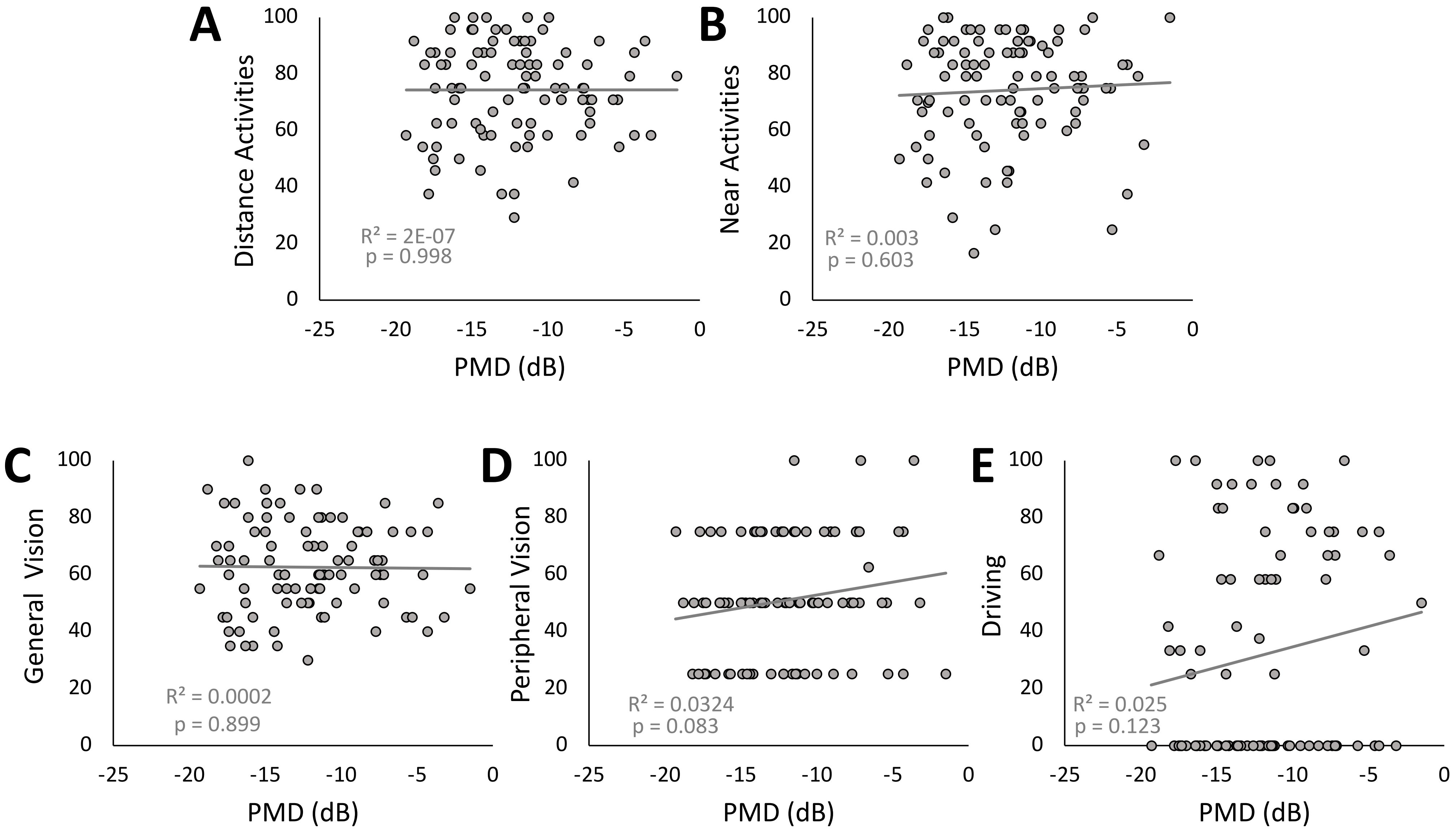

Supplementary Figure 1. Simple linear regressions correlating PMD with key subscales describing visual functioning. **A,** PMD was not significantly correlated with scores for distance activities. **B,** PMD was not significantly correlated with scores for near activities. **C,** PMD was not significantly correlated with scores for general vision. **D,** PMD was not significantly correlated with scores for peripheral vision. **E,** PMD was not significantly correlated with scores for driving.
