# Supplementary material for: Vision‐related quality of life after unilateral occipital stroke
Source: Brain Behav. 2024 Jul 2;14(7):e3582. doi: 10.1002/brb3.3582 (PMC11219293; doi:10.1002/brb3.3582)
Supplement: Supplementary file 2 — Supporting Information Table 1. Demographic characteristics across the three NCT analyzed in the present study. Other than female (F) and male (M) numbers, all data are reported as mean ± SD. [file BRB3-14-e3582-s002.docx]

| **Clinical Trial Identifier** | **# F/M** | **Age (years)** | **Time post-stroke (months)** | **Binocular PMD (dB)** | **NEI-VFQ Composite Score** | **Neuro10 Composite Score** |
| --- | --- | --- | --- | --- | --- | --- |
| NCT04798924 | 10/15 | 50.8±11.7 | 3.5±1.3 | -11.3±4.2 | 69.1±14.2 | 68.4±13.8 |
| NCT03350919 | 10/32 | 59.5±9.9 | 45.1±76.7 | -13.1±4.0 | 67.1±17.4 | 75.5±18.0 |
| NCT05098236 | 6/22 | 61.1±11.9 | 17.4±25.2 | -11.3±3.8 | 68.9±12.0 | 73.4±13.2 |

**Supplementary Table 1. Demographic characteristics across the three NCT analyzed in the present study.** Other than female (F) and male (M) numbers, all data are reported as mean ± SD.
